# Child-Pugh classification dependent alterations in serum leptin levels among cirrhotic patients: a case controlled study

**DOI:** 10.1186/1471-230X-4-23

**Published:** 2004-09-23

**Authors:** Fusun F Bolukbas, Cengiz Bolukbas, Mehmet Horoz, Mahmut Gumus, Mehmet Erdogan, Fadile Zeyrek, Ali Yayla, Oya Ovunc

**Affiliations:** 1 Department of Internal Medicine, Gastroenterology Division, Harran University, Medical Faculty, Sanliurfa, Turkey; 2 Department of Internal Medicine, Harran University, Medical Faculty, Sanliurfa, Turkey; 3 Internal Medicine Clinic, Dr.Lutfi Kirdar Kartal Training and Research Hospital, Istanbul, Turkey; 4 Department of Microbiology, Harran University, Medical Faculty, Sanliurfa, Turkey; 5 Gastroenterology Clinic, Haydarpasa Numune Training and Research Hospital, Istanbul, Turkey

## Abstract

**Background:**

As anorexia and hypermetabolism are common in cirrhosis, leptin levels may be increased in this disease. In this study, we investigated the relation between the severity of disease and serum leptin levels in post-hepatitis cirrhosis and the role of body composition, gender and viral aetiology of cirrhosis in this association.

**Methods:**

Thirty-five cases with post-hepatitis cirrhosis and 15 healthy controls were enrolled in this study. Body composition including body mass index, body fat percentage and body fat mass were determined. Serum leptin levels were assayed.

**Results:**

Leptin levels were significantly higher among cirrhotic patients independent of sex compared to controls (p = 0.001). Female patients in both groups have had higher leptin levels than males (in cirrhotics p = 0.029, in controls p = 0.02).

Cirrhotic patients in each of A, B and C subgroups according to the Child- Pugh classification revealed significantly different levels compared to controls (p = 0.046, p = 0.004, p = 0.0001, respectively). Male cirrhotics in Child-Pugh Class B and C subgroups had significantly higher leptin levels compared to male controls (p = 0.006, p = 0.008). On the other hand, female patients only in Child Pugh class C subgroup have had higher levels of serum leptin compared to controls (p = 0.022).

Child-Pugh classification has been found to be the sole discriminator in determination of leptin levels in cirrhotics by linear regression (beta: 0.435 p = 0.015).

**Conclusion:**

Serum leptin levels increase in advanced liver disease independently of gender, body composition in posthepatitic cirrhosis. The increase is more abundant among patients that belong to C subgroup according to the Child- Pugh classification.

## Background

Leptin, a 16-kilodalton protein, is involved in the regulation of food intake and body composition [1]. It was discovered in 1994 by Friedman et al. [2] and has been proposed to physiologically regulate body weight by suppressing appetite and increasing energy expenditure [1,3,4].

In normal humans, circulating level of leptin is higher in women than in men [3,5]. Besides of gender dependency, circulating leptin levels correlate with the body fat mass (BFM) and body mass index (BMI) in healthy subjects [5-7].

Malnutrition is a common feature of cirrhotic patients [8]. A negative energy balance, and thus catabolism caused by energy expenditure is considered to be of pathophysiological relevance in cirrhosis [9]. Several studies have shown that circulating leptin levels are modestly elevated in patients with alcoholic cirrhosis, suggesting that leptin might be involved in the malnutrition of cirrhosis [10,11]. While some studies have been supported these findings, others have reported low serum leptin levels in post-hepatitis cirrhotic patients [10,12,13]. In addition, nutritional status of cirrhotic cases represents a wide range in normal to severe malnutrition, connected with severity of the disease [8]. It appears that relationship of serum leptin levels and nutritional status in post-hepatitis cirrhosis has not been fully clarified yet.

In this study, we investigated the relation between the severity of disease and serum leptin levels in post-hepatitis cirrhosis and the role of body composition, gender and viral aetiology of cirrhosis in this association.

## Methods

Thirty-five cases with post-hepatitis cirrhosis (17 male, 18 female; mean age: 51.5 ± 12) which were diagnosed on the basis of the clinical, laboratory, radiological, and/or histopathological findings, and 15 healthy controls (8 male, 7 female; mean age: 49.4 ± 8) were enrolled in this study. Cirrhotic cases were assigned into 3 groups on the basis of the Child-Pugh classification [14] as follows: Child A (n = 10), Child B (n = 14) and Child C (n = 11). Causative agents of cirrhosis were viral hepatitis B (n = 20) and hepatitis C (n = 15). As leptin is a gender dependent peptide, control and cirrhotic group were divided into two groups as male and female. Exclusion criteria were history of cancer, diabetes mellitus, and alcoholism, existence of pleural effusion, gastrointestinal bleeding, acute infection and renal failure, treatment with corticosteroids, immunosuppressive agents and oral contraceptive within the last 6 months.

Control group consisted of healthy individuals with normal medical history, physical examination and blood biochemistry. None of them have had a restriction of diet for loosing weight during the last three months. Subjects who receive any medication have not been included into control group. The local human institutional review committee approved the study and written consents were received from all participants.

Body composition such as BMI, skin fold thickness, body fat percentage (BFP), and BFM analysis was performed in both cirrhotic cases and controls. To avoid incorrect BMI determination and body composition analysis, cirrhotic cases with ascite and edema had been put on sodium restricted diet of 51 mmol per day and they were received diuretics (spiranolactone 100–200 mg and, if necessary, furosemide 40–80 mg per day) until ascite and edema have been resolved. Cirrhotic cases with refractory ascite unresponsive to therapy impaired renal function following diuretic therapy, and triceps skinfold thickness less than 10^th ^percentile [15] were excluded.

BMI was determined as the actual body weight relative to the square of the body height (BMI, kg/m^2^). Measurements of skin fold thickness were conducted at four different sites on the left side of the body (triceps, biceps, sub-scapular and supra-iliac) using a Holtain skinfold caliper (Holtain, Crosswell, Crymych, Dyfed, UK). All the measurements were made by the same physician (FB). Two measurements were made at each site and the average values were obtained. The BFP was calculated using the Jacksons' formula [16]. BFM was calculated using BFP and body weight as kilogram.

Diet containing 1 g/kg body weight of protein and 30 kcal/kg body weight of non-protein calories, was described to be consumed by both cirrhotic and controls for 2 weeks before serum leptin level measurement was performed.

Blood samples were obtained in the morning following 12 hours of fasting, they were centrifugated and serum was separated after storage for one hour at room temperature. Biochemical analyses were done during the same day. Serum samples for measurement of leptin levels were stored at -20°C until they were used.

Serum leptin levels were measured as ng/ml via immunoradiometric assay (IRMA) method by using Human Leptin IRMA DSL-23100 (Diagnostic Systems Laboratories, Inc. Texas, USA) kit. Following test procedures, test tubes were assessed with Gammabyt-CR gamma counter for one hour. Measurements for standards, controls and serums were repeated for confirmation. Sensitivity of the test was 0.10 ng/ml.

### Statistical analysis

Data were presented as median and range. Qualitative variables were assessed by Chi-square test. Between whole and sub-group comparisons were performed by non-parametric Kruskal-Wallis and Mann-Whitney U tests. A linear logistic regression analysis was performed with serum leptin levels as dependent variable and age, gender, BFM, aetiology of cirrhosis, Child-Pugh classification as independent variables in cirrhotics. A p value of <0.05 was considered statistically significant.

## Results

### Patient profiles and body composition

**Figure 1:**
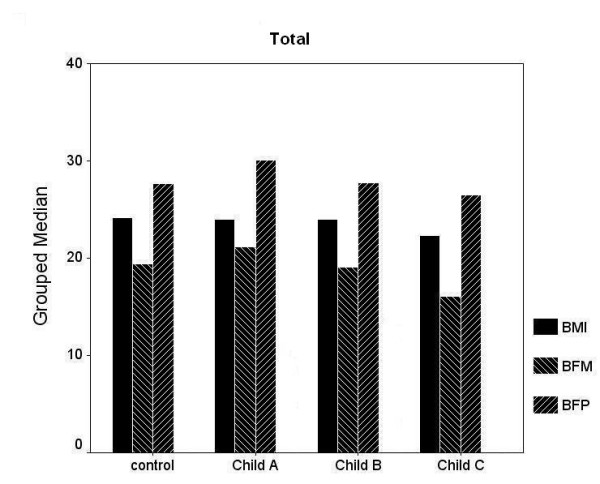
Following Child Pugh Classification, there were no significant differences in terms of body mass index (BMI), body fat percentage (BFP) and body fat mass (BFM) between controls and cirrhotic patients (both, p > 0.05).

**Figure 2:**
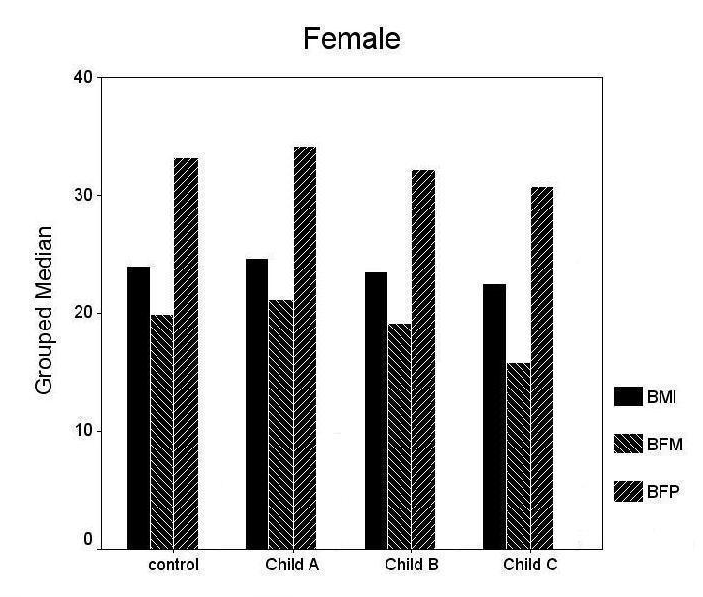
Following Child-Pugh Classification, there were no significant differences in terms of body mass index (BMI), body fat percentage (BFP) and body fat mass (BFM) between female controls and female cirrhotic patients (both, p > 0.05).

**Figure 3:**
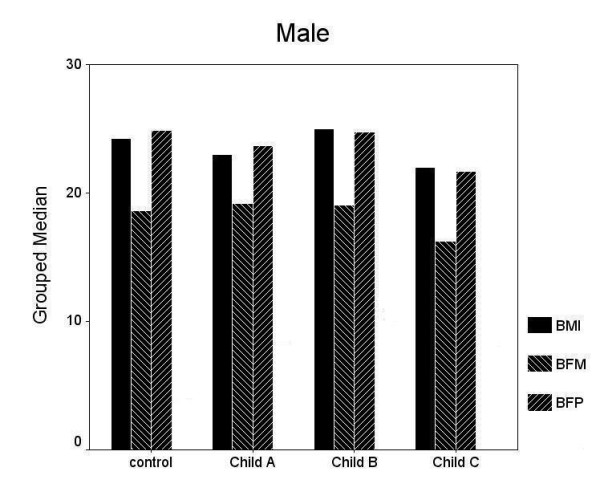
Following Child-Pugh Classification, there were no significant differences in terms of body mass index (BMI), body fat percentage (BFP) and body fat mass (BFM) between male controls and male cirrhotic patients (both, p > 0.05).

Clinical and demographic characteristics of all and gender-based sub-groups were shown in table 1. In male and female subjects, no statistically significant difference was observed in age, BMI, BFP and BFM between the controls and cirrhotic group (both, p > 0.05). Following Child Pugh Classification, gender based or not, there were no significant differences in terms of BMI, BFP and BFM between controls and cirrhotic patients in each group according to the Child-Pugh classification (Figure 1) for each sex (Figure 2 and 3).

**Table 1:** Characteristics of cirrhotic patients and controls in whole and gender based sub-groups.

	Age Year	BMI Kg/m^2^	BFP %	BFM Kg	Leptin ng/ml
Cirrhotic (n = 35)	53 (28–73)	24 (18–33)	27.9 (18.5–39)	19.4 (9.6–34)	13.5 (1.6–41)*
Female (n = 18)	48 (28–73)	24 (18–33)	32 (24–39)	20.1 (9.6–34)	15.5 (7.4–41)**
Male (n = 17)	54 (35–66)	23 (19–27)	24.6 (18.5–28)	18.6 (11–26)	10.9 (1.6–36)***
Control (n = 15)	47 (37–65)	24 (20–26)	27.7 (22.4–37)	19.4 (14–26)	6.4 (0.14–16.3)
Female (n = 7)	43 (37–61)	24 (22–25)	33.2 (30–37)	20 (18–26)	7.2 (5.58–16.3)
Male (n = 8)	53 (42–65)	24 (20–26)	24.9 (22.4–28)	18.6 (14–21)	3.7 (0.14–8.7)

Note: Data were presented as median and range.

Groups and subgroups did not differ in terms of age, BMI, BFP, and BFM (p > 0.05).

*Cirrhotic vs. controls (p = 0.001), ** cirrhotic females vs. control females (p = 0.025), ***Cirrhotic males vs. control males (p = 0.002)

BMI; Body mass index, BFP; Body fat percentage, BFM; Body fat mass

### Leptin levels

Serum leptin levels were significantly higher in cirrhotic group than controls (p = 0.001) (Table 1). There was a significant difference between the leptin levels of men and women in both control and cirrhotic groups (p = 0.029, p = 0.02, respectively) (Table 1). Leptin levels were elevated in both female and male cirrhotics compared to controls (p = 0.025, p = 0.002, respectively) (Table 1).

Cirrhotic patients in each of A, B and C subgroups according to the Child- Pugh classification revealed significantly different leptin levels [(ng/ml with median and range; 9.46 (1.6–30), 12.8 (4.2–18.8), 14.7 (8–41), respectively)] compared to controls (ng/ml with median and range; 6.4 (0.14–16.3) (p = 0.046, p = 0.004, p = 0.0001, respectively).

**Figure 4:**
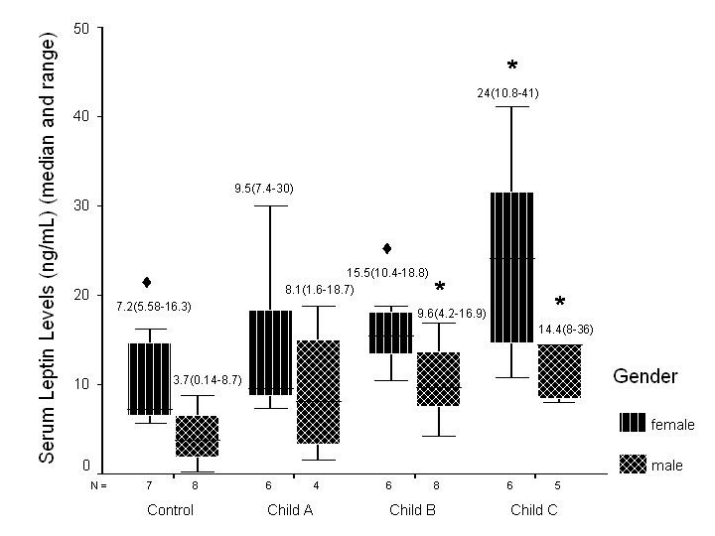
Leptin levels in controls and cirrhotic patients by gender and Child-Pugh class. Male patients in the control group had significantly lower leptin levels compared to cirrhotic male cases that belongs to B and C classes (p = 0.006, p = 0.008, respectively). On the other hand, female gender revealed significant difference only between Child Pugh C class patients and controls (p = 0.02). In controls and Child Pugh B class patients, females had higher leptin levels than males. *P < 0.02 vs. controls, in the same gender. ^◆^*P *< 0.05 vs. different gender in the same group.

Gender based serum leptin levels of controls and cirrhotic cases that were grouped according to Child Pugh Classification were as shown in Figure 4. Male patients in the control group had significantly lower serum leptin levels compared to cirrhotic male cases that belongs to B and C classes (p = 0.006, p = 0.008, respectively). However, the difference was not significant between the control males and Child Pugh class A males (p = 0.234). On the other hand, female gender revealed significant difference only between Child Pugh C class patients and controls (p = 0.022).

When age, gender, BFM, hepatitis B and C virus as etiologic factors of cirrhosis and child A, B and C as Child-Pugh classification were tested as independent variables for determination of serum leptin levels as dependent variable by linear logistic regression analysis in cirrhotic group, analysis result showed that Child-Pugh classification was the sole discriminator in determination of serum leptin levels in cirrhotic cases (beta: 0.435, p = 0.015) (Table 2).

**Table 2:** Linear regression analysis (R^2 ^= 0.326) with serum leptin as dependent variable in the cirrhotic group (n = 35).

Independent variables	Beta	p
Gender (M-F)	-0.307	0.065
Age (years)	-0.227	0.183
BFM (kg)	0.006	0.974
Viral Etiologic Factor (HBV-HCV)	0.167	0.315
Child-Pugh Classification (A-B-C)	0.435	0.015*

Beta, beta regression coefficient; M, Male; F, Female; BFM, Body fat mass; HBV, Hepatitis B Virus; HCV, Hepatitis C Virus; A, Child-Pugh Class A; B, Child-Pugh Class B; C, Child-Pugh Class C.

## Discussion

Leptin regulates body weight by suppressing appetite and increasing energy expenditure [1,3,4]. Anorexia and increased energy expenditure usually accompanies to the cirrhosis [17]. McCullough et al. reported modestly elevated circulating leptin levels in patients with alcoholic cirrhosis and they suggested that elevated serum leptin levels in cirrhosis might be responsible for the high prevalence of malnutrition among cirrhotic patients [11]. In our study, we also observed that circulating leptin levels were increased in non-alcoholic cirrhosis caused by viral hepatitis without severe energy malnutrition state.

Leptin levels are higher in woman than in men [3,6]. McCullough et al. found higher leptin levels among female cirrhotics than male cirrhotics, although the difference was not statistically significant [11]. These concepts are especially important in cirrhosis, because cirrhotics have gender-dependent alterations in body composition and sex steroids [18,19]. When we considered gender in our study, serum leptin levels were significantly higher among females than males in both controls and cirrhotics. In addition, cirrhotic females and males had higher levels of serum leptin than the controls with the same gender.

Since BMI and BFM values do not differ according to the sex and the presence or absence of cirrhosis, increased serum leptin levels could not be simply dedicated to BFM or malnutrition status in cirrhosis. In addition, linear regression test in the present study has shown that disease severity, which was determined by Child-Pugh classification, was the sole significant determinant of serum leptin levels in cirrhosis. In previous studies, association between the severity of cirrhosis and serum leptin levels is controversial [11-13]. Henriksen et al suggested that the elevated circulating leptin in patients with alcoholic cirrhosis was most likely caused by a combination of decreased renal extraction and increased release from subcutaneous abdominal, femoral, gluteal, retroperitoneal, pelvic, and upper limb fat tissue areas [20]. For this reason, we excluded cases with renal clearance impairment to avoid of accumulation of leptin in serum. In addition, using 4 different site of skinfold thickness measurement to calculate BFP and excluding cases with ascite that do not respond to diuretic therapy, we targeted to determine the relationship between body composition and serum leptin levels in controls and cirrhotics. In this study, BFM was found to associate with serum leptin levels in controls. However, BFM does not associate with serum leptin levels among cirrhotic patients. Therefore, we conclude that leptin production may differ among healthy and cirrhotic subjects.

In an animal study, it has been shown that chronic ethanol consumption leads to increased serum concentrations of tumor necrosis factor and related cytokines such as leptin by inducing over production of these factors in the liver and peripheral adipose tissues [21]. Leptin secretion from adipocytes may be enhanced by cytokines released as a part of the inflammatory or fibrogenic process. Alternatively, as suggested, cirrhotic patients may simply exhibit decreased hepatic clearance of this protein [22].

## Conclusion

Serum leptin levels increase in advanced liver disease independently of gender, body composition and viral etiologic factor in post-hepatitis cirrhosis. The increase is more abundant among patients that belong to C subgroup according to the Child- Pugh classification.

## Abbreviations

BMI, body mass index; BFP, body fat percentage; BFM, body fat mass

## Competing interests

The authors declare that they have no competing interests.

## Authors' contributions

Bolukbas FF conceived of the study, and participated in its design and coordination. Bolukbas FF, Bolukbas C, Erdogan M and Zeyrek F collected the samples and carried out the laboratory analysis. Bolukbas C conceived of the study and participated in the sequence alignment and drafted the manuscript. Horoz M participated in the design of the study, participated in the sequence alignment and drafted the manuscript. Gumus M collected the clinical data and performed the statistical analysis. Yayla A drafted the manuscript and revised it critically for important intellectual content. Ovunc O participated in study design and coordination and revised the manuscript critically for important intellectual content.

All authors read and approved the final manuscript.

## Pre-publication history

The pre-publication history for this paper can be accessed here:


